# Locally advanced cervical cancer with bladder invasion: clinical outcomes and predictive factors for vesicovaginal fistulae

**DOI:** 10.18632/oncotarget.24271

**Published:** 2018-01-18

**Authors:** Roger Sun, Ines Koubaa, Elaine Johanna Limkin, Isabelle Dumas, Enrica Bentivegna, Eduardo Castanon, Sébastien Gouy, Cynthia Baratiny, Fyo Monnot, Pierre Maroun, Samy Ammari, Elise Zareski, Corinne Balleyguier, Éric Deutsch, Philippe Morice, Christine Haie-Meder, Cyrus Chargari

**Affiliations:** ^1^ Department of Radiotherapy, Gustave Roussy Cancer Campus, Villejuif, France; ^2^ INSERM U1030 Molecular Radiotherapy, Gustave Roussy Cancer Campus, Villejuif, France; ^3^ Department of Radiology, Gustave Roussy Cancer Campus, Villejuif, France; ^4^ Department of Medical Physics, Gustave Roussy Cancer Campus, Villejuif, France; ^5^ Department of Surgery, Gustave Roussy Cancer Campus, Villejuif, France; ^6^ Department of Drug development, Gustave Roussy Cancer Campus, Villejuif, France; ^7^ Université Paris Sud, Université Paris-Saclay, Le Kremlin-Bicêtre, France; ^8^ Institut de Recherche Biomédicale des Armées, Brétigny-sur-Orge, France; ^9^ French Military Health Service Academy, Ecole du Val-de-Grace, Paris, France

**Keywords:** cervical cancer, locally advanced, brachytherapy, bladder invasion, vesicovaginal fistula

## Abstract

**Objective:**

We report outcomes of cervical cancer patients with bladder invasion (CCBI) at diagnosis, with focus on the incidence and predictive factors of vesicovaginal fistula (VVF).

**Results:**

Seventy-one patients were identified. Twenty-one (30%) had para-aortic nodal involvement. Eight had VVF at diagnosis. With a mean follow-up time of 34.2 months (range: 1.9 months–14.8 years), among 63 patients without VVF at diagnosis, 15 (24%) developed VVF. A VVF occurred in 19% of patients without local relapses (9/48) and 40% of patients with local relapse (6/15). Two-year overall survival (OS), disease-free survival (DFS) and local control rates were 56.4% (95% CI: 44.1–67.9%), 39.1% (95% CI: 28.1–51.4%) and 63.8% (95% CI: 50.4–75.4%), respectively. Para-aortic nodes were associated with poorer OS (adjusted HR = 3.78, *P*-value = 0.001). In multivariate analysis, anterior tumor necrosis on baseline MRI was associated with VVF formation (63% vs 0% at 1 year, adjusted-HR = 34.13, 95% CI: 4.07–286, *P*-value = 0.001), as well as the height of the bladder wall involvement of >26 mm (adjusted-HR = 5.08, 95% CI: 1.38–18.64, *P*-value = 0.014).

**Conclusions:**

A curative intent strategy including brachytherapy is feasible in patients with CCBI, with VVF occurrence in 24% of the patients. MRI patterns help predicting VVF occurrence.

**Methods:**

Patients with locally advanced CCBI treated with (chemo)radiation ± brachytherapy in our institute from 1989 to 2015 were analyzed. Reviews of baseline magnetic resonance imaging (MRI) scans were carried out blind to clinical data, retrieving potential parameters correlated to VVF formation (including necrosis and tumor volume).

## INTRODUCTION

Cervical cancers with bladder invasion (CCBI) classified as stage IVA according to the International Federation of Gynecology and Obstetrics (FIGO) represent approximately 2% of cervical cancers. The prognosis is poor, with an estimated 5-year overall-survival (OS) of 20% [1]. Moreover, development of vesicovaginal fistulae (VVF) is frequent, with retrospective data showing that VVF occurs in up to 50% of stage IVA cases, with no established predictive factors [2, 3].

The current standard of care for locally advanced cervical cancers consists of concurrent cisplatin-containing chemotherapy followed by brachytherapy [4–7]. However, although brachytherapy (BT) is a mainstay of treatment, data are limited on its efficacy in stage IVA disease, with some suggesting that bladder infiltration is a contraindication to brachytherapy [6, 8–14].

This study aims to report treatment outcomes in this particular situation, with focus on the incidence of and predictive factors for VVF formation.

## RESULTS

### Patients and tumors

Seventy-one patients with CCBI were identified. Forty-five (63.4%) received the totality of their treatment in our institute and 26 (36.6%) were referred only for brachytherapy, after having received external beam radiotherapy (EBRT) in other centers.

Patients’ characteristics are summarized in Table 1. Forty-eight patients (67.6%) presented with stage IVA disease, and 23 (32.4%) had extrapelvic metastases, including 18 (25.4%) with para-aortic lymph node (PALN) metastases and five (7.0%) with oligo-metastatic disease.

**Table 1:** Patients characteristics

Characteristics		No. of patients (%)
***N***		71
**Age (years)**	Mean ± SD	53.7 ± 13.7
	≤45	22 (31.0)
	46–55	18 (25.4)
	56–65	15 (21.1)
	≥ 66	16 (22.5)
**PS**	0	13 (18.3)
	1	41 (57.7)
	2	14 (19.7)
	3	3 (4.2)
**Follow-up time (months)**	Median	19.9
	Mean ± SD	34.2 ± 38.4
	Range	1.9 - 177.4
**Tumor characteristics**	Maximum tumor diameter (mm): mean ± SD	67.0 ± 21.4
	Tumor volume (cc)	112.71 ± 97.47
**Invasion of bladder**	Histologically proven	15 (21.1)
	Cystoscopy	13 (18.3)
	Laparoscopy	1 (1.4)
	MRI	39 (54.9)
	CT scan	3 (4.2)
**Invasion of rectum**		13 (18.3)
**Fistula at diagnosis**	Bladder	8 (11.3)
	Rectum	1 (1.4)
**Parametrial invasion**	Unilateral	8 (11.3)
	Bilateral	61 (85.9)
	No	2 (2.8)
	Clinical distal parametrial involvement	45 (63.4)
**Vaginal invasion**	Yes	55 (77.5)
	Limited to the upper two-third	36 (50.7)
	Lower third involvement	19 (26.8)
**Hydronephrosis**	Unilateral	37 (52.1)
	Bilateral	20 (28.2)
	No	14 (19.7)
**Stage (FIGO)**	IV A	48 (67.6)
	IV B	23 (32.4)
**Nodal involvement**	No	24 (33.8)
	Pelvic nodes only	26 (36.6)
	Para-aortic nodes only	5 (7.0)
	Both pelvic and para-aortic	16 (22.5)
**PALN involvement**	CT scan	3 (4.2)
	MRI	9 (12.7)
	PET	8 (11.3)
	PALN dissection	1 (1.4)
**Metastasis at diagnosis**	Ovarian	2 (2.8)
	Peritoneal	1 (1.4)
	Dorsal vertebra	1 (1.4)
	Supraclavicular node	1 (1.4)
**Para-aortic nodes laparoscopic staging**	Positive	1 (1.4)
	Negative	11 (15.5)
**Histology**	Squamous cell carcinoma	61 (85.9)
	Adenocarcinoma	8 (11.3)
	Other	2 (2.8)
**Differentiation**	Low	21 (29.6)
	Moderate	20 (28.2)
	High	19 (26.8)
	Unknown	11 (15.5)

Abbreviations: SD: standard deviation; FIGO: International Federation of Gynecology, Obstetrics; CT: computed Tomography; MRI: magnetic resonance imaging. PALN: para-aortic lymph nodes.

### Radiotherapy/brachytherapy characteristics

Treatments delivered are summarized in Table 2. All patients had pelvic radiotherapy, 23 had extended field (PALN) irradiation (32.4%). Fifty-seven (80.3%) received chemotherapy: 47 (66.2%) received concomitant chemotherapy (cCRT), three (4.2%) received neoadjuvant chemotherapy, seven (9.9%) received both neoadjuvant and cCRT.

**Table 2:** Characteristics of treatment

Characteristics					No. of patients (%)	
**Chemotherapy**			Neoadjuvant CT only		3 (4.2)	
			cCRT		47 (66.2)	
			Neoadjuvant + cCRT		7 (9.9)	
			No CT		14 (19.7)	
**Number of cCRT cycles**			≤ 4		40 (56.3)	
			≥ 5		24 (33.8)	
			Unknown		7 (9.9)	
**Radiotherapy: technique**			2DCRT		19 (26.8)	
			3DCRT		35 (49.3)	
			IMRT		5 (7.0)	
			Not detailed		12 (16.9)	
**Radiotherapy: fields**			Pelvic		48 (67.6)	
			Pelvic + paraaortic		23 (32.4)	
**Sequential radiation boost**			Parametrium		12 (16.9)	
			Pelvic nodes		18 (25.4)	
			Para-aortic nodes		9 (12.7)	
**Brachytherapy**			LDR		33 (46.5)	
			PDR		31 (43.7)	
			No BT		7 (10)	
**OTT (from the start of RT to the end of BT, *n* = 64)**			≤ 55 days		38 (59.4)	
			> 55 days		26 (40.6)	
			Median (range)		54 days (42–143)	
**Dosimetric Parameters**		**Mean ± SD**		**N available**	
**Radiotherapy**	Pelvic dose	44.7 ± 3.1		71	
**Brachytherapy**	Points A dose (Gy_α/β10_)	71.1 ± 9.2		45	
	TRAK (cGy.h^−1^.m^−1^)	2.0 ± 0.4		64	
	V15Gy_α/β3 (_cm^3^)	270.2 ± 78.70		63	
**HR-CTV**	Volume (cm^3^)	48.9 ± 27.6		31	
	D_90_ (Gy_α/β10_)	72.6 ± 9.7		30	
**IR-CTV**	Volume (cm^3^)	108.5 ± 50.6		31	
	D_90_ (Gy_α/β10_)	63.1 ± 5.0		30	
**Bladder**	D_2cc_ (Gy_α/β3_)	74.7 ± 6.5		30	
	ICRU (Gy_α/β3_)	72.0 ± 13.0		57	
**Rectum**	D_2cc_ (Gy_α/β3_)	66.1 ± 5.9		30	
	ICRU (Gy_α/β3_)	74.5 ± 13.9		58	
**Sigmoid**	D_2cc_ (Gy_α/β3_)	57.8 ± 6.7		30	

Abbreviations: cCRT: concurrent chemoradiation; TRAK: total reference air kerma; ICRU: International Commission Radiation Units: HR-CTV: high-risk clinical target volume; IR: intermediate-risk clinical target volume: OTT: overall treatment time; RT: radiation therapy; LDR: low dose rate; PDR: pulse dose rate; BT: brachytherapy.

After EBRT, 64/71 (90%) patients received BT boost: 2D-low dose rate brachytherapy (LDR-BT) for 33 patients, image-guided pulse-dose-rate brachytherapy (PDR-BT) for 31 patients (magnetic resonance imaging (MRI)-guided in 25 and computed-tomography (CT)-guided in six patients). Eight patients had an interstitial boost (seven with PDR-BT, one with LDR-BT). From the beginning of EBRT to the end of BT, median overall treatment time was 54 days (range:42–143). Brachytherapy characteristics are detailed in Supplementary Table 1.

Seven patients did not receive brachytherapy: two had an EBRT boost (one refused BT, one had a major VVF contra-indicating BT); two underwent completion surgery by anterior pelvectomy after 45 Gy (Supplementary Figure 1). Seven patients (10%) did not complete the radiotherapy (median dose: 39.6 Gy, range: 30-43.2): three for declining performance status, four for toxicities but the latter received brachytherapy.

### Dosimetric parameters

Dosimetric parameters are summarized in Table 2. Median point A dose was 72.1 Gy_α/β10_ (range: 30.9–88.8 Gy_α/β10_). Median International Commission on Radiation Units (ICRU) bladder point dose was 71.9 Gy_α/β3_ (range: 16.1–109.2 Gy_α/β3_) and median ICRU rectal point dose was 74.7 Gy_α/β3_ (range: 31.6–105.5 Gy_α/β3_). The treated volume (volume of 60 Gy isodose) and ICRU rectal point dose were significantly lower with PDR-BT (245.5 vs 294.1 cc, *P*-value = 0.01 and 71 vs 77.4 Gy α/β3, *P*-value = 0.02 respectively) than with LDR-BT.

For the 31 patients treated by image-guided PDR-BT, the median high risk clinical target volume (HR-CTV) and intermediate risk clinical target volume (IR-CTV) were 41.6 cm3 (range: 11.1–113.2) and 109.5 cm3 (range: 43.7–224.5), respectively. Median total doses delivered to 90% of the IR-CTV and HR-CTV were 61.75 Gy_α/β10_ (range: 55.2–76.4 Gy_α/β10_) and 70.70 Gy_α/β10_ (range: 57.80–94.90 Gy_α/β10_), respectively.

### Treatment outcome

After a mean follow-up time of 34.2 months (range: 1.9 months–14.8 years), persistent or recurrent disease was reported in 36/71 patients (50.7%). Distant failure was the most common cause of disease progression (25/71 patients [35.2%]). Pelvic lymph node failure was seen in 8/71 patients (11.2%). Local recurrences (LR) occurred in 16/71 patients (22.5%), with eight having only LR (11.3%). Thirteen of the 16 LRs and 7/8 isolated relapses were in patients who had pelvic only disease at diagnosis.

**Figure 1:**
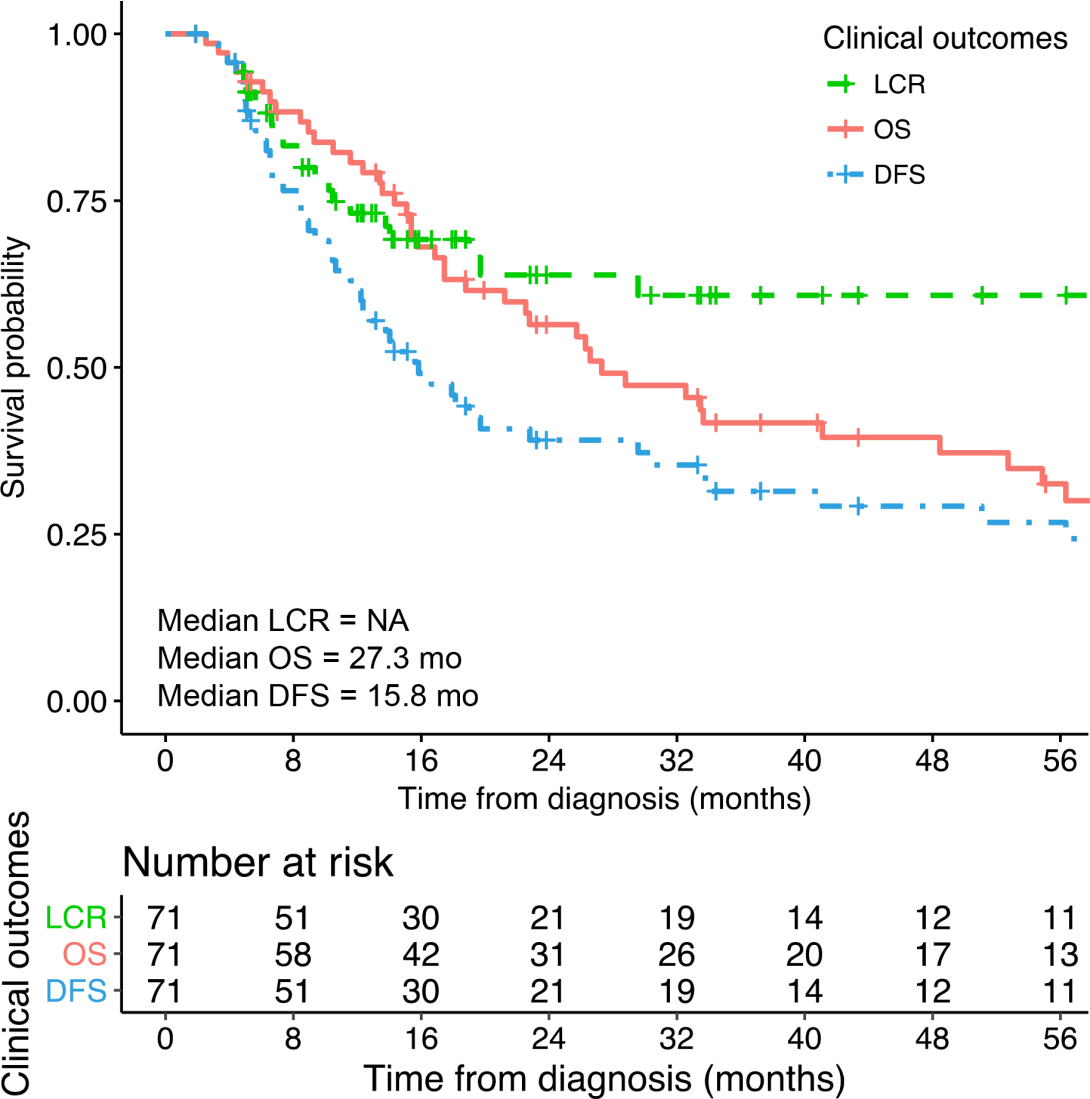
Overall survival (OS), disease free survival (DFS) times and local control rates (LCR)

At the time of analysis (October 2016), 15 patients were alive, including 14 without evidence of disease. Three other patients had stopped their follow-up after more than 10 years of follow-up. Eight patients (11%) were lost to follow up after the treatment (mean follow-up time: 235 ± 179 days). Estimated 2-year and median OS were 56.4% (95% CI: 44.1–67.9%) and 27.3 months (95% CI: 18.8-52.7 months) respectively. Estimated 2-year and median disease-free-survival (DFS) were 39.1% (95% CI: 28.1–51.4%) and 15.8 months (95% CI: 12.0–29.6 months) respectively. Local control (LC) rates were 73.1% (95% CI: 61.0–82.5%) at 1 year and 63.8% (95% CI: 50.4–75.4%) at 2 years (Figure 1).

In patients with only pelvic disease, 2-year and median OS and DFS were 68.8% (95% CI: 54.2–80.4%) and 33.6 months (95% CI: 26.3–94.2 months), and 47.1% (95% CI: 33.3–61.3%) and 19.7 months (95% CI: 14–56.3 months) respectively. LC rates at 1 and 2 years were 79.7% (95% CI: 65.6–88.9%) and 68.0% (95% CI: 52.3–80.5%).

Salvage pelvectomy was performed for three patients due to LR suspicion (Supplementary Figure 1). Histological examinations showed few tumor cells for one patient, and a complete response for a second patient. All the three patients were still alive at the last follow-up (OS: 11, 25, 40 months).

### Prognostic factors

Univariate analyses are summarized in supplementary materials (Supplementary Table 2). Multivariate analyses are provided in Table 3. In univariate analysis, PALN involvement was associated with poorer OS (median: 13.4 vs 33.6 months, HR = 3.52, 95% CI: 1.86–6.66, *P*-value < 0.001) and DFS (median: 8.5 vs 19.0 months, HR: 2.50, 95% CI: 1.37–4.55, *P*-value < 0.01), but remained significant only for OS in multivariate analysis. Similar results were seen for performance status (PS) ≥ 2 (HR = 2.00, 95% CI: 1.07–3.74, and 1.98, 95% CI: 1.07–3.68, for OS and DFS respectively) with non-significant P-values in multivariate analysis. Use of cCRT was associated with better outcomes for DFS (HR = 0.37, 95% CI: 0.20–0.68) and LC (HR = 0.29, 95% CI: 0.13–0.68), while the completion of the cCRT (≥5 cycles) was associated with better OS (HR = 0.47, 95% CI: 0.24–0.92), in univariate analysis. In multivariate analysis, pelvic dose <45 Gy, use of concomitant chemotherapy, and tumor anterior-posterior diameter of > 5.5 cm were significantly associated with poorer LC (Table 3), Height of the bladder wall involvement was associated with DFS and LC rate in univariate analysis only (HR = 2.07, 95% CI: 1.03–4.16 and HR = 3.38, 95% CI: 1.05–10.82). No relation could be drawn between points A or ICRU bladder/rectal point doses and probability of survival or LC. PDR-BT dose volume parameters could not be introduced in the model due to the limited number of patients treated with image-guided PDR-BT. VVF was not significantly associated with lower OS or DFS.

**Table 3:** Multivariate analysis of prognostic factors for overall survival, progression-free survival, local control and vesicovaginal fistula formation

**Overall survival**				
**Multivariate analysis**	**Model**	**Cox-HR**	**CI 95%**	***P*-value**
**Para-aortic nodes**	57 pts	3.78	1.75–8.19	0.001^*^
**Completion of Concomitant CT**	37 events	0.57	0.28–1.18	0.13
**Tumor ant-post diameter > 5.5 cm**		1.23	0.59–2.55	0.58
**Pelvic nodes**		0.83	0.38–1.82	0.64
**PS ≥ 2**		2.78	1.30–5.92	0.008^*^
**Progression-free survival**				
**Multivariate analysis**	**Model**	**Cox-HR**	**CI 95%**	***P*-value**
**Use of Concomitant CT**	49 pts	0.51	0.22–1.15	0.10
**Tumor ant-post diameter > 5.5 cm**	34 events	1.45	0.57–3.68	0.44
**Para-aortic nodes**		1.84	0.73–4.61	0.19
**PS ≥ 2**		2.18	0.73–6.45	0.16
**ICRU bladder point > 70 Gy**		1.55	0.72–3.31	0.26
**Local control rate**				
**Multivariate analysis**	**Model 1**	**Cox-HR**	**CI 95%**	***P*-value**
**Use of Concomitant CT**	61 pts	0.36	0.13–0.95	0.04^*^
**Tumor ant-post diameter > 5.5 cm**	19 events	3.01	1.04–8.75	0.04^*^
**Pelvic RT dose < 45 Gy**		10.88	2.92–40.40	3.6e-4^*^
**Vesicovaginal fistula formation**				
**Multivariate analysis**	**Model**	**Cox-HR**	**CI 95%**	***P*-value**
**MRI anterior necrosis**	43 pts	34.13	4.07–286	0.001^*^
**Height of bladder wall involvement > 26 mm**	13 events	5.08	1.38–18.64	0.014^*^

Abbreviations: HR: hazard ratio; CT: chemotherapy, PS: Performance Status, RVF: rectovaginal fistula, VVF: vesicovaginal fistula.

^*^Significant *P*-value in multivariate analysis.

### Urinary outcome and fistula formation

A total of 23 patients had VVF in their disease history. Eight patients had VVF prior to any treatment (11.3% of the 71 patients) and four of them without urinary symptoms received brachytherapy. VVF disappearance was observed in two of them: one at 18 Gy of RT (cystoscopy), and one six months after BT (methylene blue test). Among the 63 patients (88.7%) without VVF prior to treatment, 15/63 (23.8%) developed VVF during follow-up, with 13/15 (87%) occurring the year following the start of radiotherapy (five before and ten after BT). Median time to onset was 3.1 months after the start of EBRT (95% CI: 1.5–11.2 months). Six out of 15 VVFs (40%) were associated with local relapse, while nine patients out of the 48 patients (19%) with no VVF at diagnosis and no local relapse, developed a VVF which were therefore considered as complications.

Eleven of the 23 patients with VVF (48%) underwent surgery related to VVF: anterior pelvectomy + cutaneous uretero-ileostomy (*n* = 5), isolated cutaneous uretero-ileostomy (*n* = 3), continent ileocolonic urinary reservoir (*n* = 1), total pelvectomy (*n* = 1), VVF repair (*n* = 1). Five patients also had a colostomy for associated recto-vaginal fistulae or severe digestive symptoms. At the last follow-up, 12/23 patients (52.2%) had no urinary symptom, while five (21.7%) still experienced repeated urinary tract infections or dribbling.

### Predictive factors for VVF formation

Univariate predictive factors tested for VVF formation in the population without VVF at diagnosis (63 patients) are provided in supplementary materials (Supplementary Table 3). Results of multivariate analysis are detailed in Table 3 and Supplementary Table 4.

**Figure 2:**
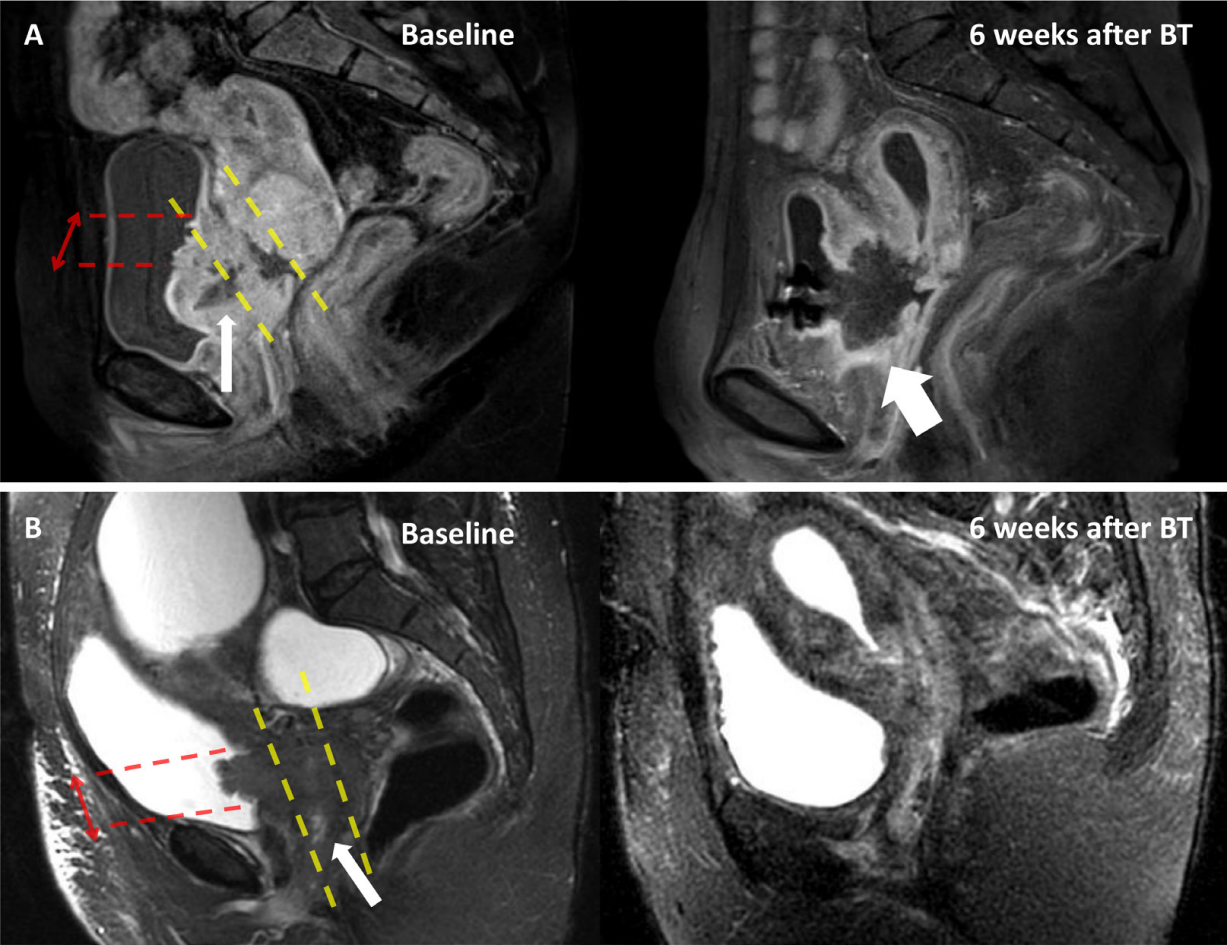
Example of anterior necrosis leading to VVF Magnetic Resonance Imaging (MRI) exams of two patients with cervical cancer stage IVA (patient A: contrast enhanced T1-weighted MRI and patient B: T2-weighted MRI) at baseline (left), and 6 weeks after brachytherapy (right). Patient A had a tumor necrosis (thin arrow) which involved the anterior third of the tumor, while there was no anterior necrosis for patient B. Six weeks after brachytherapy (BT), patient A developed a vesicovaginal fistula (thick arrow), while patient B had a complete response. Red arrows: height of the bladder involvement. Yellow dashed line: delimit the anterior-third, mid-third and posterior-third of the tumor.

**Figure 3:**
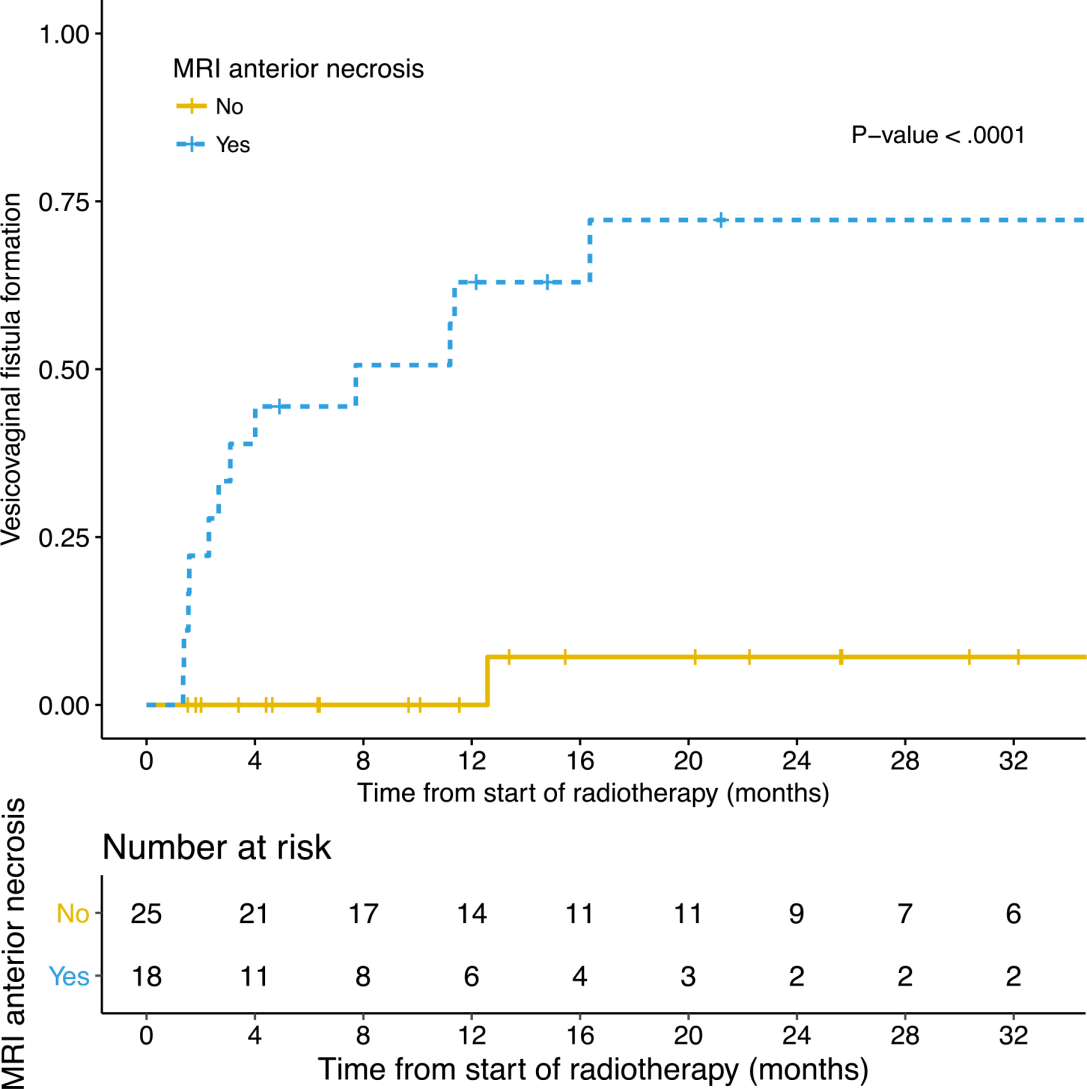
Rate of VVF formation according to the presence of MRI anterior necrosis

Baseline MRIs were available for 43/63 (68%) of patients without VVF at diagnosis. Anterior tumor necrosis was present in 18 patients, of which 12 developed VVF (Figure 2). Among the 25 patients without anterior tumor necrosis, one developed a VVF. Anterior tumor necrosis was significantly associated with the occurrence of VVF (63% vs 0% at one year, HR = 22.45, 95% CI: 2.91–173.32, *P*-value = 1e-05) (Figure 3). Patients for whom the height of the bladder wall involvement was higher than the median of 26mm, seemed to have earlier onset of VVF at one year (45% vs 16% at one year, HR = 2.66, 95% CI: 0.87–8.20, *P*-value = 0.08) (Supplementary Figure 2). In a multivariate analysis taking into account anterior necrosis and height of the bladder wall involvement, both variables were significant (Table 3).

## DISCUSSION

CCBI is infrequent and data are scarce, generally showing poor prognosis and high local failure rates. This study with 71 patients, although retrospective, is to date the largest monocentric cohort assessing the clinical outcomes in this population, and one of the first examining prognostic factors associated with VVF [15, 2, 3, 16, 17]. Patient prognosis was poor, with median OS and DFS of 27.3 and 15.8 months, respectively. Approximately half of patients experienced tumor progression, distant relapses being the main cause of failure, and local recurrences affecting 16/71 (22.5%) patients. These results are consistent with previously published data, reporting median OS and DFS of 21.2 and 10.1 months and 3year-OS and DFS of 47% and 28%, respectively [15, 16]. In contrast, a study of 34 patients with stage IVA cervical cancers reported a median OS of 49 months [17]. This difference could be explained by a large number of patients with stage IVB in our cohort (32%). LC was nevertheless similar compared to previous retrospective studies of IVA cervical cancer, with local failure rates ranging from 39 to 44% [15, 17].

Prognostic factors for tumor control and survival were identified in this subset of patients. PALN involvement was an independent poor prognostic factor for OS. Use of cCRT was a favorable prognostic factor in univariate, but not in multivariate analysis for OS and DFS, and remained significant in multivariate analysis for LC. Likewise, Moore *et al*. also reported a positive trend for survival benefit with chemotherapy in patients with stage IVA disease [2]. The median survival was 8.9 months for patients with stage IVA disease treated with radiation alone (*n* = 5) versus 22.6 months for patients treated with cCRT (*n* = 16), although this was not statistically significant [2]. The non-significance can be explained by the low number of patients in both studies. Moreover, the meta-analysis by Green *et al*. suggests that stage I–II patients benefit the most from cCRT for OS (*P*-value = 0.009 for trials randomizing ≥70% of stage I–II) [18]. The same trend was found in the Cochrane meta-analysis [19]. As for our results, the lower benefit of cCRT on OS may be due to the high incidence distant relapses, suggesting the importance of increasing not only local but also systemic treatment. Two international ongoing phase III studies aim to assess the survival benefit of additional chemotherapy delivered either before (the INTERLACE trial, NCT01566240) or after cCRT and brachytherapy (the OUTBACK trial, NCT01414608) for IB1-node positive to IVA disease.

The limited size of our cohort did not allow us to find any association between survival and 3D-dosimetric parameters, but application of GEC-ESTRO guidelines [20–22] may improve the quality of radiotherapy and brachytherapy as suggested by Pötter *et al*, reporting an increase in OS and LC for tumors >5cm since the advent of MRI-based treatment planning, in an analysis of 145 patients with cervical cancer stages IB–IVA [23].

One major concern in patients receiving brachytherapy for stage IVA cervical cancer is the risk of VVF. It is frequently advocated that bladder involvement should be a contra-indication to brachytherapy because of a high risk of VVF formation. In this series wherein most patients received brachytherapy despite bladder involvement, we reported an acceptable rate of VVF. The 24% rate of VVFs occurring during or after treatment was comparable to the 22% reported in two previous studies where majority of patients received brachytherapy [3, 24] although this estimation is variable in literature, ranging from <10% [15–17, 25] to approximately 50% [2]. Tumor necrosis on baseline MRI, particularly anterior necrosis, was strongly associated with VVF formation, which occurred earlier when the height of the bladder wall involvement was high. If confirmed in further studies, such as the multicenter study EMBRACE (https://www.embracestudy.dk/), these MRI findings could be used for stratifying patients. So far, due to the lack of large cohorts, no strong predictive factor was identified. Moore *et al.* suggested that smoking was predictive of VVF formation in univariate analysis (73% vs 27%, *P*-value = 0.03, among 11 patients with VVF), but this was not confirmed either by Biewenga *et al*. (HR 2.2 95% CI: 0.4–13, *P*-value = 0.39) or by our results [2, 3].

The main limitation of our study is its retrospective nature, with patients being treated over a large time interval, with consequent technique evolutions, leading to a non-negligible amount of missing data (mainly baseline imaging and LDR-BT dosimetric parameters). Moreover, all patients did not have histological confirmation of bladder invasion and those with clinical symptoms of VVF before any treatment were not considered for brachytherapy.

However, our data provide strong argument for the use of brachytherapy as part of the definitive treatment of selected CCBI. Brachytherapy is the best modality for dose escalation, paramount in this subset of patients with high frequency of local relapses. The incidence of VVF in patients who did not experience local relapse was acceptable; furthermore, majority of surviving patients had no urinary symptoms at their last follow-up [12–14]. The finding that 40% of patients with local relapses developed VVF emphasizes the need for aggressive and ideal treatment, including cCRT and optimal brachytherapy, which has been shown to be an independent favorable prognostic factor for OS in locally advanced cervical cancer [8]. Although the predictive value of MRI regarding occurrence of VVF need to be confirmed in a prospective cohort, knowledge of such factors may allow giving patients a more accurate information about VVF risk and associated symptoms.

To conclude, a curative intent strategy including brachytherapy as part of local treatment is feasible in patients with bladder invasion, with VVF formation in 24% of them. MRI has a strong predictive value for VVF occurrence. Since prognosis remains poor, intensification of local and systemic therapies should be considered.

## MATERIALS AND METHODS

### Patient selection

Clinical and dosimetric data of patients with CCBI treated in our Institute between 1989 and 2015 were retrospectively reviewed. Stage IVA was defined by a histologically proven bladder involvement by biopsy, a visual confirmation of bladder involvement by cystoscopy, and/or an unequivocal bladder involvement at CT or MRI or cystoscopy according to the radiologist. Patients with a stage IVA disease and treated with (chemo)radiation ± brachytherapy were included. Patients with PALN involvement (suspected on imaging or confirmed after PALN dissection), or stage IVB oligo-metastatic disease who received local treatment with curative intent were also included. This retrospective study was conducted in accordance with ethical standards and with the 1964 Helsinki declaration and its later amendments.

### EBRT

All patients received pelvic EBRT. Para-aortic irradiation was used if PALN metastases were suspected by imaging. From 2007, PALN dissection was performed on patients without para-aortic positron emission tomography-computed tomography (PET-CT) uptake to guide EBRT fields, based on retrospective data showing a high incidence of false negative PET-CT results in the para-aortic area [26–28].

EBRT was delivered using 1.8–2 Gy daily fractions, five fractions per week, with a total dose of 45 Gy. Two-dimensional conventional radiotherapy was used from 1989 to 2003, and 3D conformal radiotherapy was used since 2004. From 2014, patients receiving EBRT in our institution were treated with intensity-modulated radiotherapy. cCRT consisted of weekly cisplatin (40 mg/m^2^/week) or weekly carboplatin AUC2 (in case of renal contra-indication), except in cases of patient’s refusal or comorbidity.

A sequential or synchronous EBRT boost was given to deliver a total dose of 60 Gy to macroscopically involved nodes (pelvic or PALN), taking into account the contribution of brachytherapy.

### Brachytherapy

After EBRT, patients without symptoms of VVF were candidates for an utero-vaginal brachytherapy boost, following a previously reported procedure [29–33]. LDR was delivered with Cesium-137 sources before 2006. Thereafter, PDR with Iridium-192 stepping source was used.

Treatment planning was based on radiographs or 3D imaging (CT or MRI), depending on the year of treatment. For radiograph-based planning, dosimetry was based on orthogonal radiographs and 15 Gy was prescribed to the isodose that encompassed the cervix and residual tumor with a 0.5–1 cm margin in all three planes, without exceeding ICRU 38 dose constraints. For 3D treatments, tumor volumes and organs at risk (OARs) were delineated on T2–weighted MRI or CT scan. The aim was to deliver ≥60 Gy to 90% of the IR-CTV, defined according to the Brachytherapy Group of the European Society of Therapeutic Radiology and Oncology (GEC-ESTRO) guidelines, without exceeding dose constraints to the most irradiated D2cm^3^ of rectum, bladder and sigmoid colon [20, 34].

For PDR treatments, continuous hourly pulses were delivered, 24 h/day. For both LDR and PDR treatments, a dose rate of 0.6 Gy/h to ICRU points or to the most irradiated D 2cm^3^ of OARs was not exceeded. Interstitial needles were applied when necessary to improve tumor coverage, especially in the parametria. Contribution of interstitial catheters did not exceed 20% of the Total Reference Air Kerma (TRAK).

Doses delivered were reported after conversion into 2 Gy/fraction radiobiological dose equivalents, using the linear-quadratic model (repair half-time: 1.5 h, α/β ratio = 10 Gy and 3 Gy for target volumes and OARs, respectively [35].

### Surgery

Post-radiation hysterectomy was discussed for patients who could not receive brachytherapy boost due to clinical symptoms of VVF, had residual disease 6–8 weeks after brachytherapy completion, or experienced local recurrence during follow-up. If indicated, surgery was an anterior pelvectomy +/− urinary diversion. Patients with VVF and not indicated for BT boost could also receive an EBRT boost.

### Follow-up

Patients were evaluated weekly during radiotherapy. Follow-up was ensured at 6 weeks following BT, then every 3 months for 2 years, every 6 months until year 5, then annually thereafter. MRIs were performed systematically 6 weeks after treatment completion, then every 6 months. Failures were classified according to the site of first tumor relapse and defined as centropelvic (cervix, uterine corpus, vagina, parametrium, bladder), regional (pelvic nodes), or distant (PALN or visceral).

### Statistical analysis

Potential prognostic and predictive factors of VVF were examined from medical charts, and included tumor and treatment related variables (see Supplementary Materials), as well as detailed radiological characteristics of the tumor: volume, height of bladder wall involvement, tumor necrosis (high signal intensity on T2-weighted MRI, low signal with no enhancement on contrast-enhanced T1-weighted MRI) and localization (“anterior” if involving the anterior third of the tumor) (Figure 2). Radiological parameters were double-blind assessed by R.S and I.K. In case of disagreement, imaging was assessed by a senior radiologist (S.A).

Wilcoxon tests and fisher tests were used for comparisons between variables. LC rate, DFS and OS were computed according to the Kaplan–Meier method and Cox’s proportional-hazards survival estimates. For continuous variable analyses, median was used to separate patients into two groups. Follow-up and survival times were calculated from the date of histo-pathological diagnosis. Endpoint was any death for OS, local recurrence for LC, and any recurrence for DFS (including all-cause deaths for DFS, and death related to disease before any relapse for LC). Time to VVF formation was estimated from the start of radiotherapy.

*P*-values were estimated using double-sided tests. A threshold of <.05 was defined for significance. Non-redundant variables with a *P*-value of <.1 were included in multivariate analyses. Statistical analyses were carried out using R version 3.3.2 [36] (http://www.R-project.org) and “survival” R-package (version 2.40–1 [37]).

## SUPPLEMENTARY MATERIALS FIGURES AND TABLES



## Abbreviations

AUC: area under the curve
BT: brachytherapy
CCBI: cervical cancer patients with bladder invasion
cCRT: concomitant chemotherapy
CI: confidence interval
CT: computed tomography scan
EBRT: external beam radiotherapy
FIGO: International Federation of Gynecology and Obstetrics
GEC-ESTRO: Group of the European Society of Therapeutic Radiology and Oncology
HR: hazard-ratio
HR-CTV: high risk clinical target volume
I.C.R.U: International Commission on Radiation Units
IMRT: intensity-modulated radiotherapy
IR-CTV: intermediate risk clinical target volume
LDR: low dose rate brachytherapy
LC: local control rate
LR: local recurrence
MRI: magnetic resonance imaging
OAR: organ at risk
OS: overall survival
PALN: para-aortic lymph node
PDR: pulse dose rate brachytherapy
PET-CT: Positron emission tomography-computed tomography
DFS: disease-free survival
RT: radiotherapy
VVF: vesicovaginal fistula.
