# Supplementary material for: Locally advanced cervical cancer with bladder invasion: clinical outcomes and predictive factors for vesicovaginal fistulae
Source: Oncotarget. 2018 Jan 18;9(10):9299–310. doi: 10.18632/oncotarget.24271 (PMC5823628; doi:10.18632/oncotarget.24271)
Supplement: Supplementary file 1 [file oncotarget-09-9299-s001.pdf]

Locally advanced cervical cancer with bladder invasion: clinical outcomes and predictive factors for vesicovaginal fistulae

SUPPLEMENTARY MATERIALS

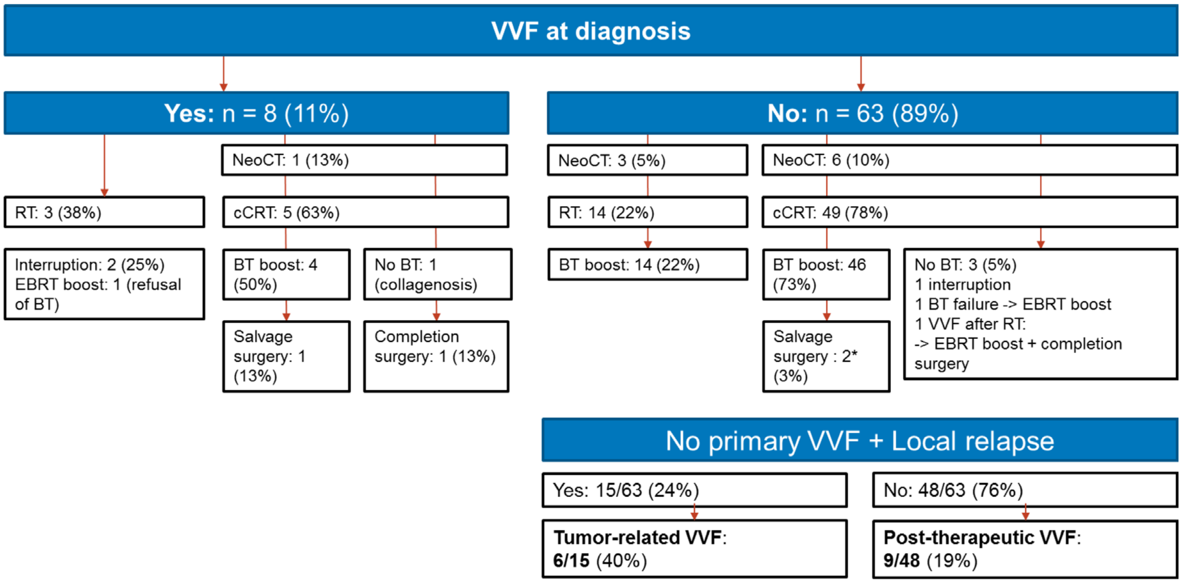

* Both patients had VVF formation and suspicion of local recurrence but one had finally a complete pathological response

Supplementary Figure 1: Flowchart of patient according to the presence or the formation of vesicovaginal fistulae.

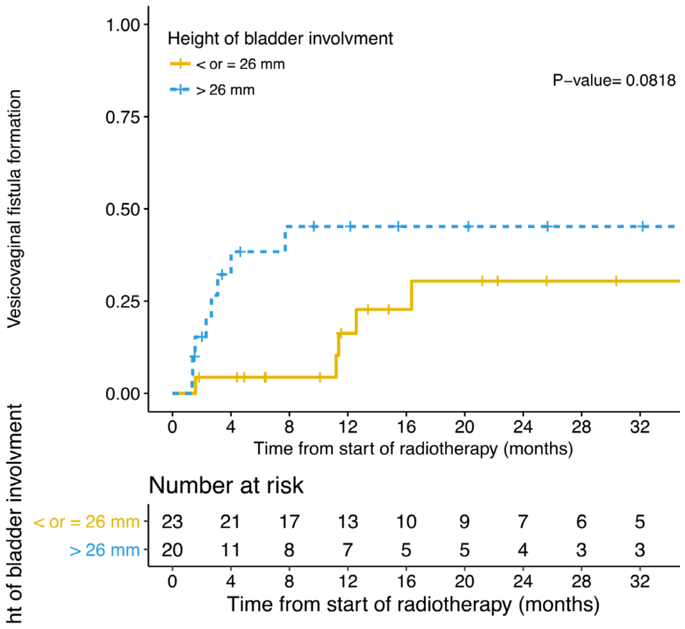

Univariate Cox P-value

Supplementary Figure 2: Rate of VVF formation according to the height of bladder wall involvement.

Supplementary Table 1: Brachytherapy characteristics according to the technique

Characteristics					
**Brachytherapy**	**LDR**		**PDR**		
Nb of patients	33 (46.5%)		31 (43.7%)		
Point A Dose	Mean ± SD	69.9 ± 13.4	Mean ± SD	71.8 ± 5.9	
Duration of treatment	Mean ± SD	3.2 ± 3.6 days	Mean ± SD	5.5 ± 5.4 days	
	n	Length of sources (mm)	*n*	Length of activation	Pulses
Intrauterine	32	Median: 60<br>Range: 24–88	31	Median: 55<br>Range: 30–95	Nb of pulses:<br>Median: 50<br>Range: 14–75
Right vaginal	33	Median: 32<br>Range: 32–48	31	Median: 30<br>Range: 20–55	Dose by pulse (cGy):<br>Median: 27<br>Range: 20–37
Left vaginal	32	Median: 32<br>Range: 32–48	31	Median: 25<br>Range: 20–55	
	LDR			PDR	
**Interstitial Brachytherapy**	Unilateral<br>Bilateral	1 (3.0%)<br>0		Unilateral<br>Bilateral	3 (9.7%)<br>4 (16.1%)
	Number of source	1		Number of probe	Range: 1–4
	Length	30		Length	Median: 27.5 mm<br>Range: 15–95 mm
				Number of pulses	Median: 34<br>Range: 29–56
				Dose by pulse (cGy)	Median: 27<br>Range: 25–35

Supplementary Table 2: Prognostic factors for survival and local control (univariate analysis)

			OS			PFS			LCR		
	**Tested factor**	**N pts**	**N event**	**Cox– HR**	***P*–value**	**N event**	**Cox HR**	***P*–value**	**N event**	**Cox HR**	***P*–value**
Age	>55 yrs	35/71	21/45	0.83	.54	23/49	0.83	.51	8/22	0.53	.14
**PS**	≥ 2	17/71	15/45	2.00	.04*	15/49	1.98	.04*	7/22	1.80	.22
Pathology	NSCC	10/71	7/45	1.66	.25	7/49	1.37	.46	3/22	1.12	.85
Tobacco	Yes	20/70	12/44	0.87	.69	13/48	0.78	.43	4/21	0.52	.21
ANC	>7.5G/l	17/42	11/28	0.87	.72	12/30	0.81	.57	6/14	1.11	.84
WBC	>10G/l	16/43	10/29	0.88	.75	11/31	0.85	.67	5/15	0.91	.87
Hb	<11	25/46	20/31	1.45	.31	21/33	1.36	.39	7/15	0.67	.44
Bladder invasion histo/cysto–proven	No	42/71	25/45	0.96	.89	28/49	0.94	.84	13/22	1.02	.97
Pelvic nodes	Yes	42/71	29/45	1.83	.05*	30/49	1.38	.28	11/22	0.75	.50
**Para–aortic nodes**	Yes	21/71	16/45	3.52	<.001*	17/49	2.50	.005*	8/22	2.27	.08
MRI based volume	>80 cc	21/61	16/39	1.47	.25	17/43	1.34	.36	9/19	1.91	.16
**Tumor longest diameter**	>6 cm	35/61	24/39	1.09	.79	26/43	0.94	.84	11/19	0.86	.74
**Tumor ant–post diameter**	>5.5 cm	31/61	23/39	2.00	.04*	26/43	2.39	.01*	13/19	2.82	.03*
**Height of the bladder wall involvement**	>26 mm	24/49	17/30	1.60	.20	19/33	2.07	.04*	10/14	3.38	.03*
MRI anterior necrosis	Yes	23/49	12/30	0.57	.13	15/33	0.68	.28	8/14	1.14	.80
MRI necrosis	Yes	32/49	18/30	0.64	.24	21/33	0.77	.48	10/14	1.11	.87
Hydronephrosis (bilat vs no or unilat)	Bilat	20/71	16/45	1.51	.20	16/49	1.32	.38	7/22	1.30	.57
Parametrial invasion (bilat vs no or unilat)	Bilat	61/71	39/45	0.97	.95	42/49	0.88	.77	18/22	0.74	.59
VVF at any moment	Yes	23/71	15/45	1.16	.80	17/49	1.15	.65	11/22	2.15	.08
**RVF at any moment**	Yes	8/71	5/45	0.92	.91	7/49	1.87	.16	6/22	3.95	.01*
Overall time of RT	>37 days	20/71	16/45	0.95	.43	17/49	1.59	.14	9/22	2.00	.12
Pelvic RT dose	<45Gy	7/71	4/45	1.29	.38	4/49	1.41	.53	4/22	4.19	.03*
Neoadjuvant CT	Yes	10/71	6/45	1.65	.69	6/49	0.71	.41	3/22	0.87	.82
**Concomitant CT**	Yes	54/71	30/45	0.84	.04*	32/49	0.37	.002*	12/22	0.29	.01*
**Completion of Concomitant CT**	Yes	24/64	12/42	0.50	.02*	13/46	0.45	.01*	6/22	0.50	.13
Brachytherapy	Yes	64/71	40/45	0.47	.41	44/49	0.82	.69	18/22	0.36	.10
Interstitial probes	Yes	7/64	3/40	0.60	.37	3/44	0.50	.20	1/18	0.40	.31
PDR vs LDR	PDR	31/64	16/40	0.66	.68	19/44	0.91	.76	8/18	0.82	.68
OTT	>55 days	26/64	18/40	0.87	.50	20/44	0.99	.97	7/18	0.77	.59
HR–CTV volume	>40 cc	17/31	10/16	0.81	.52	12/19	1.29	.60	5/8	1.43	.62
HR–CTV D90	<80 Gy	24/30	13/15	0.70	.99	15/18	0.81	.74	6/8	0.55	.49
IR–CTV volume	>100 cc	16/31	8/16	1.01	.47	10/19	1.26	.62	5/8	2.02	.33
IR–CTV D90	<60 Gy	9/30	5/15	0.68	.77	6/18	0.81	.67	2/8	0.55	.45
Points A dose	>70 Gy	26/45	17/25	0.85	.52	18/28	1.30	.51	8/11	1.98	.29
ICRU bladder point	>70 Gy	33/57	24/34	1.32	.25	26/38	1.86	.07	10/16	1.44	.47
ICRU rectal point	>70 Gy	33/58	23/35	1.54	.70	25/39	1.22	.55	9/16	0.97	.95
Bladder D2cc	>75 Gy	15/30	8/15	1.15	.17	10/18	1.73	.25	4/8	1.35	.68
Sigmoid D2cc	>60 Gy	9/30	4/15	2.12	.47	6/18	1.29	.62	4/8	2.61	.18
Rectum D2cc	>65 Gy	15/30	8/15	0.66	.45	9/18	1.27	.63	3/8	0.69	.61

NSCC: non–squamous cell carcinoma, ANC, WBC, Hb: absolute neutrophil count, white blood cells count and hemoglobin concentration at treatment start, VVF, RVF: vesico–, recto–vaginal fistulae, OTT: overall treatment time, HR–CTV: high–risk clinical target volume, IR–CTV: intermediate risk clinical target volume; OS: overall survival; PFS: progression–free survival: LCR: local control rates; *P*–values < 0.1 in bold. *: Significant *P*–values.

Supplementary Table 3: Prognostic factors of vesicovaginal fistula formation, and associated risk of formation at 1 year from the start of radiotherapy (univariate analysis)

Variables	Tested factor	N pts	N event	Risk of VVF at 1yr from the start of radiotherapy (%)		Cox–HR	CI 95%	*P*–value
				Without	With factor			
**Age**	>55 yrs	32/63	5/15	29	18	0.40	0.14–1.17	0.08
PS	≥2	14/63	3/15	22	28	0.87	0.25–3.1	0.83
Pathology	NSCC	8/63	2/15	24	17	1.04	0.23–4.63	0.96
Tabaco	Yes	18/63	4/15	25	18	0.80	0.25–2.5	0.69
ANC	>7.5 G/l	13/36	5/10	20	32	2.21	0.64–7.66	0.22
WBC	>10 G/l	14/37	5/10	19	30	2.12	0.61–7.38	0.24
Hb	<11	21/40	6/11	17	31	1.17	0.36–3.85	0.79
Bladder invasion histo/ cysto–proven	No	37/63	10/15	23	23	1.58	0.54–4.63	0.39
Pelvic nodes	Yes	37/63	7/15	25	22	0.67	0.24–1.86	0.44
Para–aortic nodes	Yes	19/63	3/15	24	23	0.75	0.21–2.7	0.65
**MRI based volume**	>80 cc	21/42	3/12	37	17	0.32	0.09–1.19	0.07
Tumor longest diameter	>6 cm	30/54	8/14	18	31	1.08	0.38–3.13	0.88
Tumor ant–post diameter	>5.5 cm	27/54	7/14	20	31	1.22	0.43–3.5	0.71
Height of the bladder wall involvement	>26 mm	20/43	8/13	16	45	2.66	0.87–8.20	0.08
**MRI anterior necrosis**	Yes	18/43	12/13	0	63	22.45	2.91–173.32	1.10–5
**MRI necrosis**	Yes	26/43	12/13	0	45	9.03	1.17–69.55	<0.01
Hydronephrosis	Bilat	16/63	5/15	18	39	1.68	0.57–4.93	0.36
Parametrial invasion	Bilat	53/63	14/15	10	26	3.15	0.41–23.95	0.19
**RVF at any moment**	Yes	7/63	5/15	18	62	4.83	1.63–14.26	0.01
Overall time of RT	>37 days	18/63	3/15	26	17	0.57	0.16–2.01	0.35
Pelvic RT dose	<45 Gy	5/63	1/15	23	20	1.26	0.16–9.61	0.83
Neoadjuvant CT	Yes	9/63	3/15	21	38	1.57	0.44–5.58	0.50
Concomitant CT	Yes	49/63	11/15	31	21	0.69	0.22–2.19	0.54
Completion of Concomitant CT	Yes	22/57	8/15	23	29	1.62	0.58–4.47	0.35
Interstitial probes	Yes	7/60	2/14	23	17	1.30	0.29–5.84	0.74
PDR vs LDR	PDR	29/60	7/14	23	22	1.03	0.36–2.95	0.95
OTT	>55 days	26/60	6/14	22	22	0.87	0.3–2.5	0.79
**HR–CTV volume**	>40 cc	16/29	6/7	8	32	4.72	0.57–39.23	0.09
HR–CTV D90	<80 Gy	23/28	6/7	25	22	1.15	0.14–9.66	0.89
**IR–CTV volume**	>100 cc	15/29	6/7	8	36	6.42	0.77–53.66	0.04
IR–CTV D90	<60 Gy	8/28	2/7	22	25	0.98	0.19–5.04	0.98
Points A dose	>70 Gy	23/41	5/9	24	13	0.92	0.25–3.41	0.90
ICRU bladder point	>70 Gy	32/53	4/10	25	16	0.40	0.11–1.41	0.15
ICRU rectal point	>70 Gy	31/54	5/11	23	20	0.63	0.19–2.07	0.45
**Bladder D2cc**	>75 Gy	14/28	1/7	36	9	0.15	0.02–1.23	0.03
**Sigmoid D2cc**	>60 Gy	9/28	5/7	11	47	7.17	1.37–37.43	0.01
Rectum D2cc	>65 Gy	15/28	4/7	17	28	1.43	0.32–6.4	0.64

NSCC: non–squamous cell carcinoma, ANC, WBC, Hb: absolute neutrophil count, white blood cells count and hemoglobin concentration at treatment start, VVF, RVF: vesico–, recto–vaginal fistulae, OTT: overall treatment time, HR–CTV: high–risk clinical target volume, IR–CTV: intermediate risk clinical target volume.
P–values < .01 in bold. *: Significant *P*–values.

Supplementary Table 4: Prognostic factors of vesicovaginal fistula formation, other multivariate models

Vesicovaginal fistula formation				
**Multivariate analysis**	**Model**	**Cox–HR**	**CI 95%**	***P*–value**
**MRI anterior necrosis**	43 pts<br>13 events	18.20	2.2–148	0.007*
**RVF at any moment**		1.85	0.58–5.85	0.30

Vesicovaginal fistula formation				
**Multivariate analysis**	**Model**	**Cox–HR**	**CI 95%**	***P*–value**
**MRI anterior necrosis**	43 pts<br>13 events	20.82	2.7–163	0.004*
**Age ≥ 55 years**		0.72	0.22–2.37	0.59

Vesicovaginal fistula formation				
**Multivariate analysis**	**Model**	**Cox–HR**	**CI 95%**	***P*–value**
**MRI anterior necrosis**	42 pts<br>12 events	18.60	2.4–211	0.006*
**MRI based volume > 80 cc**		0.63	0.17–2.38	0.49
